# The Significance of Serum HER2 Levels at Diagnosis on Intrinsic Subtype-Specific Outcome of Operable Breast Cancer Patients

**DOI:** 10.1371/journal.pone.0163370

**Published:** 2016-10-05

**Authors:** Moo Hyun Lee, So-Youn Jung, Sun Hee Kang, Eun Jin Song, In Hae Park, Sun-Young Kong, Young Mee Kwon, Keun Seok Lee, Han-Sung Kang, Eun Sook Lee

**Affiliations:** 1 Center for Breast Cancer, Research Institute and Hospital, National Cancer Center, Goyang, Gyeonggi, Republic of Korea; 2 Department of Surgery, Keimyung University, School of Medicine, Daegu, Republic of Korea; 3 Department of Laboratory Medicine, Center for Diagnostic Oncology, Research Institute and Hospital, National Cancer Center, Goyang, Republic of Korea; University of South Alabama Mitchell Cancer Institute, UNITED STATES

## Abstract

**Purpose:**

This study evaluated the association of serum HER2 (sHER2) levels at diagnosis with clinicopathologic parameters and disease free survival (DFS) in operable breast cancer patients according to intrinsic subtype.

**Methods:**

The sHER2 levels were measured using a chemiluminescence immunoassay. The HER2 status in all tumor tissues was determined by immunohistochemistry, and confirmed in equivocal cases by fluorescence in situ.

**Results:**

There were 436 consecutive stage I-III breast cancer patients with sHER2 result at diagnosis between Nov 2004 and Dec 2011. High sHER2 levels (≥ 15 ng/ml) were reported in 52 patients (11.9%) and HER2 overexpression in tumor tissue was observed in 111 patients (25.5%). High sHER2 levels were associated significantly with advanced stage (*P* < 0.001), mastectomy (*P* = 0.012), neoadjuvant chemotherapy (*P* < 0.001), anti-HER2 therapy (*P* < 0.001) and hormone therapy (*P* = 0.022). The patients with high sHER2 levels had a worse DFS (*P* < 0.001). In multivariate analysis, high sHER2 levels were associated significantly with worse DFS (HR = 2.25, 95% CI 1.27–3.99, *P* = 0.005). High sHER2 levels were associated with worse DFS in the HR+/HER2-, HR+/HER2+ and HR-/HER2+ subtypes (*P* = 0.043, 0.003 and 0.041, respectively).

**Conclusions:**

These results show that the sHER2 level at diagnosis is a useful prognostic factor in patients with operable breast cancer, especially in the HR+/HER2-, HR+/HER2+ and HR-/HER2+ subtypes.

## Introduction

The *Her-2/neu* proto-oncogene is located on chromosome 17q21 and encodes a transmembrane glycoprotein. Approximately 15% to 30% of breast cancer tumors show over-expressed human epidermal growth factor receptor 2 (HER2) protein as a result of gene amplification. Overexpression or amplification of HER2 is associated with poor prognosis [1]. Since trastuzumab, a humanized monoclonal antibody specific for HER2, became available as a treatment for HER2-positive breast cancer, HER2 status has become important in deciding whether to treat with trastuzumab [2]. To determine a patient’s HER2 status, immunohistochemistry (IHC) and fluorescence in situ hybridization (FISH) have been used. Both require a tissue sample, which is not always available. Further, IHC has relatively low accuracy, and FISH is a costly procedure.

The HER2 protein is composed of three domains: a cytoplasmic domain, a transmembrane domain and an extracellular domain. The extracellular domain of HER2 can be cleaved from the surface of breast cancer cells by matrix metalloproteases [3] and released into the serum, where it is detectable using a monoclonal antibody directed against the extracellular domain of the HER2 antigen. Serum human epidermal growth factor receptor 2 (sHER2) levels can be quantified and detectable after removing the tumor. The sHER2 levels have been associated with tumor burden [4–6], prognosis in metastatic breast cancer [7–11] and prediction of response to neoadjuvant chemotherapy [12, 13].

Although some studies demonstrated that high sHER2 levels at diagnosis are associated with poor prognosis in patients with operable breast cancer [14–16], there were few data regarding the significance of sHER2 levels at diagnosis according to each intrinsic subtype of tumor in patients with operable breast cancer. We evaluated the association between sHER2 levels at diagnosis and clinicopathologic parameters, and the correlation of sHER2 levels at diagnosis with clinical outcome according to intrinsic subtype in patients with operable breast cancer.

## Materials and Methods

### Study population

Patients were selected via an initial review of the medical records of stage I-III breast cancer patients with available results for sHER2 level at diagnosis treated between November 2004 and December 2011 at the National Cancer Center, Korea. The clinical and pathological data of 436 patients in total were reviewed. The research protocol was approved and the requirement of written informed consent was waived by the Institutional Review Board of the National Cancer Center, Korea (NCC2014-0049).

### Immunohistochemistry and fluorescence in situ hybridization

The HER2 IHC was determined using rabbit polyclonal antibodies (Dako, Produktionsvej, Glostrup, Denmark). HER2 overexpression was defined as IHC 3+ membrane staining (HER2-positive), while IHC 1+ and 0 were treated as HER2-negative. FISH was performed when the HER2 IHC scores were equivocal (2+). FISH was performed using the PathVysion Kit (Abbott Molecular, Illinois, USA), and *HER2* gene amplification was defined as *HER2/CEN* 17 ratio of ≥ 2.0. Whenever FISH tests results were available, they took precedence over the IHC results. For tumors without FISH result despite an equivocal IHC score, the HER2 status was treated as negative.

### Serum HER2 levels assay

Serum samples were obtained before surgery or before neoadjuvant chemotherapy was administered. Baseline sHER2 levels were determined with the automated ADVIA Centaur (Bayer Healthcare, Tarrytown, NY, USA), using two monoclonal antibodies directed against the extracellular domain of the HER2 antigen and direct chemiluminescent technology. Intraassay coefficient of variation for the control ranged from 3.65% to 4.38%. The cutoff value for this assay for low versus high sHER2 levels was 15 ng/ml on the basis of previous studies [14, 17–19].

### Statistical analysis

All statistical analyses were performed using STATA version 10 (StataCorp LP, College Station, TX, USA). Disease free survival (DFS) was defined as the time from the date of diagnosis to the date of first appearance of disease or death from disease. The results for sHER2 levels were analyzed as a dichotomous variable (high ≥ 15 ng/ml versus low < 15 ng/ml). Differences in categorical variables were analyzed using chi-squares test and differences in continuous variable were analyzed using Student’s t-test. DFS curves were estimated using the Kaplan-Meier method, and were compared using the log-rank test. The cox proportional hazards model was used to evaluate the effect of sHER2 levels and clinicopathologic variables on DFS. The impact of the sHER2 level on DFS also was analyzed for each intrinsic subtype based on tissue HER2 status and hormone receptor (HR) status. A two-sided p value of < 0.05 was considered statistically significant.

## Results

### Patient characteristics

Of 436 patients with a result for sHER2 at diagnosis, 103 (23.6%) had clinical stage I, 158 (36.2%) had clinical stage II, and 175 (40.1%) had clinical stage III breast cancer. The median sHER2 level at diagnosis was 9.4 ng/ml (range, 5.4–160.1), with 384 (88.2%) having low sHER2 levels (< 15 ng/ml) and 52 (11.9%) had high sHER2 levels (≥ 15 ng/ml). All patients underwent surgery: breast conserving surgery was carried out in 295 (67.7%) patients and mastectomy in 141 (32.3%) patients. A total of 186 (42.7%) patients received neoadjuvant chemotherapy. Of these, 57 (13.1%) were treated with additional adjuvant chemotherapy after surgery. Of 250 patients without neoadjuvant chemotherapy, 201 (46.1%) underwent adjuvant chemotherapy. Of 111 (25.5%) HER2-positive patients, 84 (19.3%) underwent anti-HER2 therapy. Hormone therapy was delivered to 335 (76.8%) patients and radiotherapy was delivered to 376 (86.2%) patients (Table 1).

**Table 1:** Clinicopathologic characteristics (*n* = 436).

	*n* (%)
Clinical stage	
I	103 (23.6)
II	158 (36.2)
III	175 (40.1)
Histologic grade	
1	42 (9.6)
2	192 (44)
3	153 (35.1)
Unknown	49 (11.2)
Hormone receptor status	
Negative	86 (19.7)
Positive	350 (80.3)
HER2 status	
Negative	325 (74.5)
Positive	111 (25.5)
Surgery	
Conservation	298 (67.7)
Mastectomy	141 (32.3)
Chemotherapy	
No	49 (11.2)
Adjuvant alone	201 (46.1)
Neoadjuvant +/- adjuvant	186 (42.7)
Anti-HER2 therapy	
No	352 (80.7)
Yes	84 (19.3)
Hormone therapy	
No	101 (23.2)
Yes	335 (76.8)
Radiation therapy	
No	60 (13.8)
Yes	376 (86.2)

### Relationship between sHER2 and clinicopathologic variables

High sHER2 levels were associated with advanced clinical stage (*P* < 0.001), mastectomy (*P* = 0.012), neoadjuvant chemotherapy (*P* < 0.001), anti-HER2 therapy (*P* < 0.001) and hormone therapy (*P* = 0.022). No statistical relationship was found between sHER2 levels and the other variables including histologic grade (*P* = 0.115), HR status (*P* = 0.094), radiation therapy (*P* = 0.402) and Ki-67 (*P* = 0.337). High sHER2 levels were observed in 38.7% (43 of 111) of patients with tissue HER2-positive tumor versus 2.8% (9 of 325) of patients with HER2-negative tumors. The sensitivity of sHER2 levels for predicting tissue HER2 status was 38.7%, specificity was 97.2%, positive predictive value was 82.7% and negative predictive value was 82.3% (Table 2). The sHER2 levels were moderately concordant with tissue HER2 status (82.3%, κ statistic = 0.44).

**Table 2:** Relationship between serum HER2 levels and clinicopathologic characteristics.

	Serum HER2 levels		*P*
	Low (%)	High (%)	
Clinical stage			
I	101 (26.3)	2 (3.8)	<0.001
II	149 (38.8)	9 (17.3)	
III	134 (34.9)	41 (78.8)	
Histologic grade			
1–2	216 (56.2)	18 (34.6)	0.115
3	133 (34.6)	20 (38.5)	
Unknown	35 (9.1)	14 (26.9)	
Hormone receptor status			
Negative	71 (18.5)	15 (28.8)	0.094
Positive	313 (81.5)	37 (71.2)	
Surgery			
Conservation	268 (69.8)	27 (51.9)	0.012
Mastectomy	116 (30.2)	25 (48.1)	
Neoadjuvant chemotherapy			
No	241 (62.8)	9 (17.3)	<0.001
Yes	143 (37.2)	43 (82.7)	
Anti-HER2 therapy			
No	330 (85.9)	22 (42.3)	<0.001
Yes	54 (14.1)	30 (57.7)	
Radiation therapy			
No	55 (14.3)	5 (9.6)	0.402
Yes	329 (85.7)	47 (90.4)	
Ki-67 (mean, %)	16.1	19.1	0.337
Tissue HER2 status			
Negative	316 (82.3)	9 (17.3)	<0.001
Positive	68 (17.7)	43 (82.7)	

### Survival analysis

**Fig 1:**
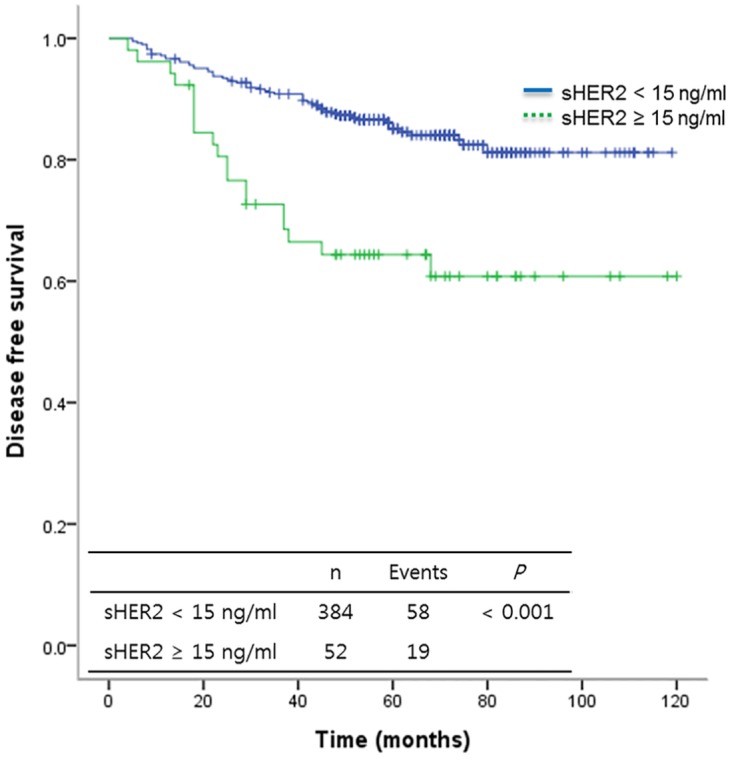
Disease free survival according to serum HER2 levels. sHER2 = serum HER2 levels.

**Fig 2:**
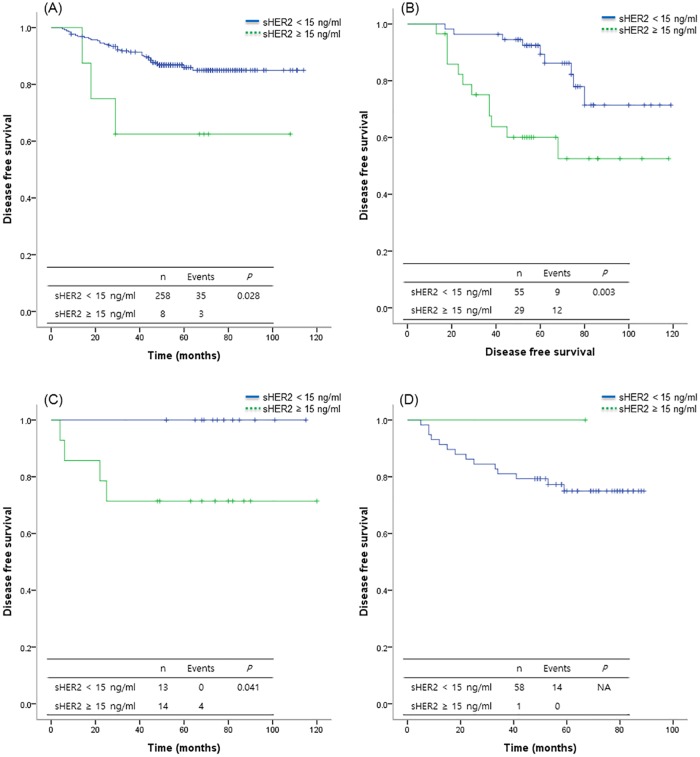
Intrinsic subtype specific disease free survival according to serum HER2 levels. (A) HR+/HER2- subtype; (B) HR+/HER2+ subtype; (C) HR-/HER2+ subtypes; (D) HR-/HER2- subtypes. sHER2 = serum HER2 levels; NA = not available.

At a median follow-up of 50.5 months (range, 9–120 months), 77 patients (17.7%) had experienced a recurrence. Locoregional recurrence was observed in 25 (32.5%) and distant metastasis in 52 (67.5%) patients. The five-year overall survival rate was 95.8%. Twenty-nine patients died with recurrence of breast cancer and one patient died from an unknown cause. The overall survival of patients with high sHER2 levels was significantly worse compared with that of patients with low sHER2 levels (*P* < 0.008) (S1 Fig). The five-year DFS rate was 88.9%. The DFS of patients with high sHER2 levels also was significantly worse when compared with that of patients with low sHER2 levels (*P* < 0.001) (Fig 1). In Multivariate analyses, high sHER2 levels (HR = 2.11, 95% CI 1.20–3.72, *P* = 0.009), clinical stage III (HR = 4.94 95% CI 2.80–8.70, *P* < 0.001) and histologic grade 3 (HR = 2.06, 95% CI 1.26–3.37, *P* = 0.004) were associated with significantly worse DFS (Table 3). DFS was analyzed for patients according to tissue HER2 status (S2 Fig). High sHER2 levels were associated with worse DFS compared with low sHER2 levels in patients with HER2-positive tumor (*P* < 0.01). Although patients with high sHER2 levels (≥ 15 ng/ml) tended to have shorter DFS in the HER2-negative tumor subgroups, the difference was not statistically significant (*P* = 0.111). DFS was analyzed according to intrinsic subtype (Fig 2). High sHER2 levels were associated with a worse DFS in HR+/HER2-, HR+/HER2+ and HR-/HER2+ subtypes (*P* = 0.028, 0.003 and 0.041, respectively).The significance of correlation between sHER2 levels and DFS in the HR-/HER2- subtype could not analyze because only one patient had increased sHER2 levels.

**Table 3:** Multivariate analysis for disease free survival.

	Hazard ratio	95% CI	*P*
Clinical stage (III vs I-II)	6.28	3.29–12.00	<0.001
Histologic grade (3 vs 1–2)	1.82	1.09–3.03	0.022
Hormone receptor status	1.22	0.63–2.35	0.528
Serum HER2 levels ≥ 15	2.25	1.27–3.99	0.005

CI = confidence interval.

## Discussion

Although the clinical importance of sHER2 levels has been studied extensively in metastatic breast cancer, there are only a few reports regarding the significance of sHER2 levels at diagnosis in the patients with operable breast cancer. Several studies have shown that sHER2 levels were strongly associated with tumor burden [4–6]. Krainer et al. reported that elevated sHER2 levels were observed in 14.2% of patients with operable breast cancer, and in 42.8% of those with stage IV disease [4]. Garoufali et al demonstrated that the mean sHER2 level in advanced disease was significantly higher than that in early disease (34.5 ng/ml vs 9.4 ng/ml) [6]. Our study showed that high serum HER2 levels were associated with advanced clinical stage, which reflects the tumor burden of patients.

In this study, elevated sHER2 levels were observed in 11.9% of operable breast cancer patients. The sensitivity of sHER2 for tissue HER2 status was 38.7% and the specificity was 97.2%. The results of sHER2 levels were moderately concordant with tissue HER2 status by IHC or FISH (82.3%). In previous studies, elevated sHER2 levels were observed in 4.4–14.2% of operable breast cancer patients [4, 14, 15, 18, 20, 21]. Ludovini et al. reported that the sensitivity of sHER2 levels for tissue HER2 status was 38.1% and the concordance rate was 87.1%, similar to our results [18].

Molina et al studied the utility of tumor markers (CEA, CA 15.3 and sHER2 levels) in 503 untreated patients with breast cancer and multivariate analysis of DFS showed CEA but not CA 15.3 and sHER2 levels were independent prognostic factors [20]. Willsher et al studied the clinical significance of sHER2 levels of patients with all stages of breast cancer [22]. Elevated sHER2 levels were detected in 15% (7/46) of stage I/II patients and 26% (9/35) of stage III patients. The sHER2 levels in stage I/II (*P* = 0.002) and stage III (*P* = 0.04) predicted a worse survival. Two studies showed significant association between high sHER2 levels and worse DFS in patients with operable breast cancer [15, 18]. Similar to the previous studies, our study also revealed that high sHER2 levels were associated with worse DFS. In multivariate analysis, sHER2 level was an independent prognostic factor with clinical stage and histologic grade. Several studies have demonstrated that the proteolytic cleavage of HER2 results in the release of the extracellular domain of HER2 and the production of truncated cell-associated fragments, and supported the hypothesis that the truncated HER2 protein is associated with enhanced signaling activity and confers an adverse prognosis [11, 23–26].

Moreno-Aspitia et al showed that high sHER2 levels were associated with poor prognosis in patients with early stage, HER2-positive breast cancer [14]. In the present study, high sHER2 levels were associated with worse DFS in the HER2 positive group. There are few reports thus far regarding the significance of sHER2 levels in HER2-negative breast cancer. Our results showed that high sHER2 levels had a tendency toward poor DFS in HER2-negative group, but this trend did not reach statistical significance (*P* = 0.111).

Our patient population was categorized into four intrinsic subtypes of HR+/HER2-, HR+/HER2+, HR-/HER2+ and HR-/HER2- according to HR status and tissue HER2 status, and the association of sHER2 with DFS was analyzed. High sHER2 levels were associated with worse DFS in the HR+/HER2- subtype as well as the HR+/HER2+ and HR-/HER2+ subtypes. Of 268 patients with HR+/HER2- tumors, only 8 patients had increased sHER2 levels. It was hard to conclude whether sHER2 status was a useful prognostic factor in the HR+/HER2- subtype because of low number of patients with increased sHER2 levels. However, there are several implications from this result. The first is the relationship between tumor volume and sHER2 levels. Of 8 patients with increased sHER2 and an HR+/HER2- tumor, 6 patients had clinical stage III disease and relapse was observed only in these patients. The next implication relates to resistance to hormone therapy in breast cancer with elevated sHER2 levels. Lipton et al reported that the poor response of the hormonal therapy is related to the increased serum HER2 in HER2-overexpressing breast cancer [27]. The mechanism of the resistance to hormone therapy is often described as crosstalk between HER2 and HR signaling [28]. In patients with both HR- and HER2-positive breast cancer, there is a need for treatment that blocks all biologic effects of both proteins. Another remarkable point is the IHC results of HER2 in these 8 patients (S1 Table). Of 8 patients, 5 had IHC 2+ and 3 of these 5 had a recurrence. The remaining 3 patients with IHC results of 0 or 1+ had no recurrence. Although all recurrence occurred in patients with advanced stage disease, patients with IHC 2+ tumors and elevated sHER2 levels, even if the patient had negative tissue HER2 status, can be necessary to consider an anti-HER2 therapies such as trastuzumab. Currently, the randomized NSABP B-47 trial is being conducted to evaluate the effects of adjuvant trastuzumab in lymph node-positive or high-risk lymph node negative, HER2-negative breast cancer patients. We will able to obtain additional information of improvement in outcome provided by trastuzumab for HR+/HER2- patients with high sHER2 levels. In the HR- / HER2- subtype, sHER2 levels were not associated with DFS and likely due to the small number of patients with elevated sHER2 levels in this group. In the HER2-negative group, sHER levels had no significant correlation with prognostic outcome, and the result may have been influenced by results of the HR-/HER- subtype. It can be speculated that the role of sHER2 levels may be different between the HR+/HER2- and HR-/HER2- subtypes.

One limitation of our study is that we used retrospectively collected data. Furthermore the small sample size at the time of recurrence makes it difficult to draw absolute conclusions about whether sHER2 levels are correlated with survival for patients with HER2-negative breast cancer including the HR+/HER2- and HR-/HER2- subtypes. In addition, we didn’t check sHER2 levels for all operable breast cancer patients, therefore, the patients included this study were not consecutive. The proportion of stage III patients was relatively high because sHER2 levels assay tended to be considered for patients with advanced stage. Finally, for tumors with equivocal IHC scores without FISH results, the HER2 status was treated as negative. However, for all patients of HR+/HER2- subtype with high sHER2 levels and equivocal IHC results, FISH results were obtained. In the HR+/HER2- subtype, the association between sHER2 level and DFS would not be affected by this factor.

## Conclusions

The sHER2 level is a useful prognostic factor for operable breast cancer patients. Elevated sHER2 levels are associated with poor DFS in the HR+/HER2-, HR+/HER2+ and HR-/HER2+ subtypes. More aggressive treatment including anti-HER2 therapy is required for patients with high sHER2 levels in the HR+/HER2- subtype as well as the HR+/HER2+ and HR-/HER2+HER2- subtype. Additional studies are needed to further evaluate the role of sHER2 assay for tissue HER2-negative tumor, including the HR+/HER2- and HR-/HER2-subtype of breast cancer using a larger clinical cohort.

## Supporting Information

S1 Fig Overall survival according to serum HER2 levels. sHER2 = serum HER2 levels.
(TIF)

S2 Fig Disease free survival according to serum HER2 levels in HER2-positive tumors and HER2-negative tumors. sHER2 = serum HER2 levels.
(TIF)

S1 Table Characteristics of HR+/HER2- subtype with high serum HER2 level. (DOCX)
