# Supplementary figures and images for: The Significance of Serum HER2 Levels at Diagnosis on Intrinsic Subtype-Specific Outcome of Operable Breast Cancer Patients
Source: PLoS One. 2016 Oct 5;11(10):e0163370. doi: 10.1371/journal.pone.0163370 (PMC5051717; doi:10.1371/journal.pone.0163370)

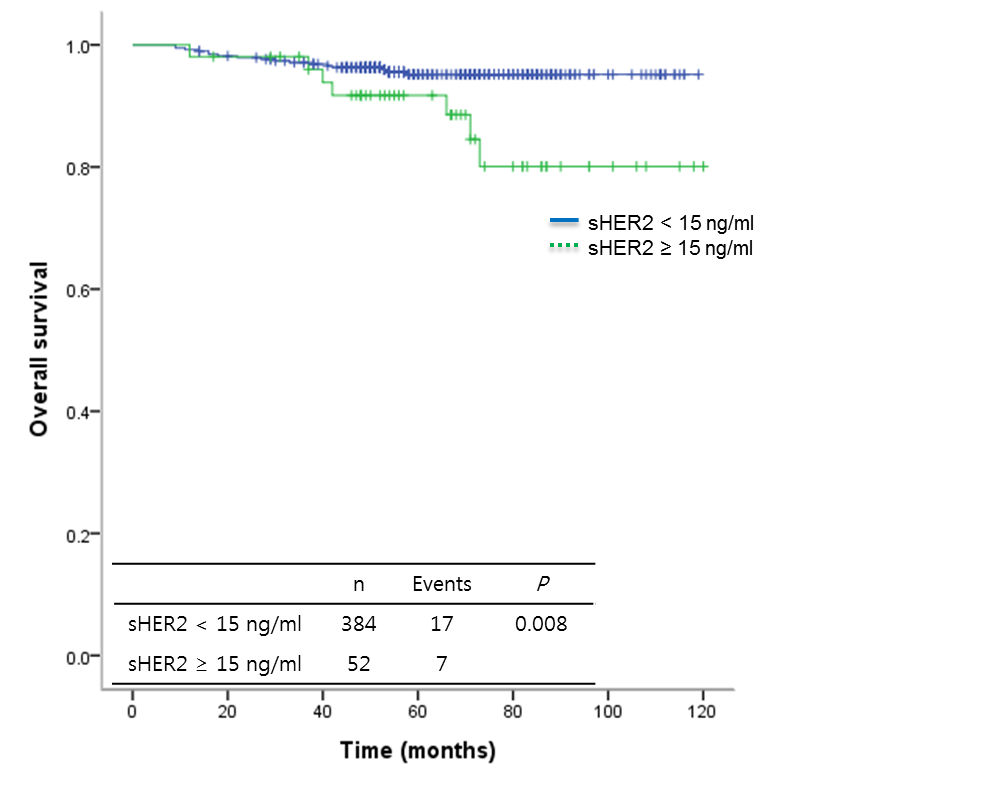

Supplement: S1 Fig — sHER2 = serum HER2 levels. (TIF) [file pone.0163370.s001.tif]

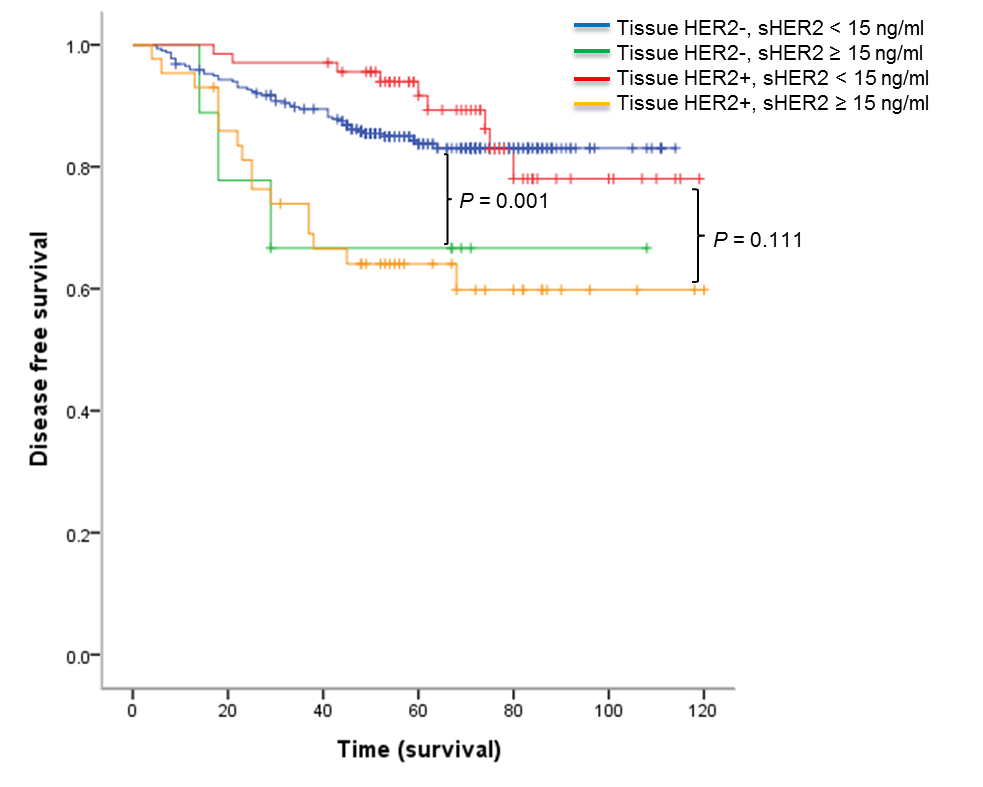

Supplement: S2 Fig — sHER2 = serum HER2 levels. (TIF) [file pone.0163370.s002.tif]
