# Supplementary material for: The Significance of Serum HER2 Levels at Diagnosis on Intrinsic Subtype-Specific Outcome of Operable Breast Cancer Patients
Source: PLoS One. 2016 Oct 5;11(10):e0163370. doi: 10.1371/journal.pone.0163370 (PMC5051717; doi:10.1371/journal.pone.0163370)
Supplement: S1 Table — (DOCX) [file pone.0163370.s003.docx]

**S1 Table. Characteristics of HR+/HER2- subtype with high serum HER2 level**

| No | NAC | Clinical stage | IHC | FISH | Ki-67(%) | sHER2(ng/ml) | HTx | Recurrence |
| --- | --- | --- | --- | --- | --- | --- | --- | --- |
| 1 | Y | III | 2+ | - | 60 | 15.5 | No | Neck node |
| 2 | Y | III | 2+ | - | 10 | 33.1 | Yes | Bone |
| 3 | Y | III | 2+ | - | 70 | 18.5 | Yes | Lung |
| 4 | Y | III | 2+ | - | 20 | 17.1 | Yes | N |
| 5 | Y | III | 1+ | NA | 10 | 20.3 | Yes | N |
| 6 | Y | III | 0 | NA | NA | 17 | Yes | N |
| 7 | N | I | 2+ | - | 10 | 16.6 | Yes | N |
| 8 | N | I | 1+ | NA | 5 | 16.3 | Yes | N |

No = patient number, NAC = neoadjuvant chemotherapy; IHC = immunohistochemistry; sHER2 = serum HER2 levels; HTx = hormonal therapy; NA = not available
